# Cancer Communication and Family Caregiver Quality of Life

**DOI:** 10.3390/bs7010012

**Published:** 2017-03-02

**Authors:** Elaine Wittenberg, Tami Borneman, Marianna Koczywas, Catherine Del Ferraro, Betty Ferrell

**Affiliations:** City of Hope Comprehensive Cancer Center, 1500 E. Duarte Road, Duarte, CA 91010, USA; tborneman@coh.org (T.B.); mkoczywas@coh.org (M.K.); cdelferraro@coh.org (C.D.F.); bferrell@coh.org (B.F.)

**Keywords:** cancer, communication, oncology, family caregiving, caregiver, quality of life

## Abstract

Family caregivers have enormous communication responsibilities tied to caregiving, such as sharing the patient’s medical history with providers, relaying diagnosis and prognosis to other family members, and making decisions about care with the patient. While caregiver stress and burden has been widely documented in the caregiving literature, little is known about how communication burden, real or perceived communication challenges, impacts caregiver quality of life. In family caregiving, the City of Hope (COH) Quality of Life model proposes that the caregiving experience is reciprocal to the patient experience, impacting physical, social, psychological, and spiritual quality of life. We used data from a pilot study testing a communication coaching call intervention with family caregivers of lung cancer patients to analyze caregiver reported communication burden and quality of life. We found variances in each quality of life domain, suggesting that caregiver interventions should range from self-care skill building for physical care to psycho-educational interventions that support caregiver coping and communication skill building. These findings demonstrate the importance of caregiver assessment and attention to communication burden in quality cancer care.

## 1. Introduction

Family caregivers of lung cancer patients have challenging communication roles related to caregiving responsibilities [1]. Caregivers are often tasked with sharing news of the diagnosis, a complex role that involves making decisions about what information should be shared, when to share it, whether they or the patient should give the news, and how the news should be shared [2]. During treatment, disagreements between patient and caregiver can occur about treatment side effects and benefits as well as what to report to physicians [3]. Lung cancer caregivers may be further challenged with patient discussions about continued tobacco use [1]. Communication dilemmas occur between patient, family, and healthcare providers when the family caregiver struggles to meet the collective interests of others by keeping them informed yet maintain individual interests by withholding information about the patient or themselves [4]. These dilemmas arise when a caregiver reluctantly serves as a family caregiver to the patient (either because there are no other family members present or others are unwilling), when caregivers must make patient care decisions but are uninformed about the patient’s wishes, or when collaboration with other family members is necessary yet family members do not have a history of collaboration. Difficult communication circumstances create communication burden [5,6], a concept we define as real or perceived communication challenges, and may impact caregiver quality of life.

Quality of life concerns of both patient and caregiver should be addressed throughout the cancer trajectory. The City of Hope Quality of Life (QOL) model [7] depicts four domains: physical, psychological, social, and spiritual as priorities for assessment to ensure comprehensive, holistic quality care. In a large, randomized controlled trial that included a tailored education intervention [8], caregivers identified patient fatigue, worry, fear, anger, cognitive changes, communication, advance directive planning, purpose and meaning, hope, and inner strength as the most common topics. While many of these topics are dependent on the caregiver’s ability to convey these needs in order to receive support, there is little understanding of the impact of these quality of life domains and communication. Understanding communication burden in the caregiving experience could inform the design of communication interventions, potentially enhancing caregiver quality of life and decreasing stress. The goal of this study was to use the Quality of Life model to examine the four quality of life domains so as to aid in the future development of customized communication interventions.

## 2. Materials and Methods

The qualitative findings from a pilot study testing the acceptability and feasibility of a nurse-delivered communication coaching call intervention are presented here. The intervention was designed to improve caregiver confidence in communication with the patient, family members, and healthcare providers. During the call, the research nurse asked the caregiver the following: Who is the most challenging to talk to about cancer (the patient, family members, or healthcare providers)? What is the most challenging for you to talk about? When you think about having to talk about cancer, and you know it will be challenging, how does this impact you physically? Psychologically? Socially? Spiritually?

The intervention protocol was based on the City of Hope Quality of Life (QOL) model and was intended to explore how communication dilemmas impact family caregivers. The intervention protocol did not change over the course of data collection and was delivered by the same nurse to all caregivers. Data were collected from consenting caregivers of lung cancer patients in an outpatient setting of a comprehensive cancer center and was approved by the hospital’s institutional review board.

### 2.1. Participants

Eligibility criteria included age 18 years of age or older, English-speaking, and primary family caregiver as identified by the lung cancer patient.

### 2.2. Procedure

Caregivers were approached by the research nurse during a routine clinic visit, an explanation of the study provided and written consent was obtained. After completing baseline questionnaires measuring psychological distress and confidence in communication, a date and time were set for the research nurse to call the following week to conduct the coaching call. Caregivers were given a copy of *A Communication Guide for Caregivers* © (2016, COMFORT Communication Project) [9] and asked to read the guide before the following week’s call. Calls were digitally recorded and served as the corpus of data for this study.

### 2.3. Data Analysis

Transcripts from the digitally recorded discussions were analyzed by two coders over two research team meetings. Caregiver responses to each quality of life domain were grouped according to a direct qualitative approach [10], and grouped data was inductively analyzed using an iterative process of theme analysis [11]. Open coding was first used to identify chunks of text suggesting a theme, themes were collapsed or associated, and interpretive claims about each domain were identified through discussion within the research team. Through this process, we discerned themes within each quality of life domain. Demographic data were summarized using SPSS.

## 3. Results

**Figure 1:**
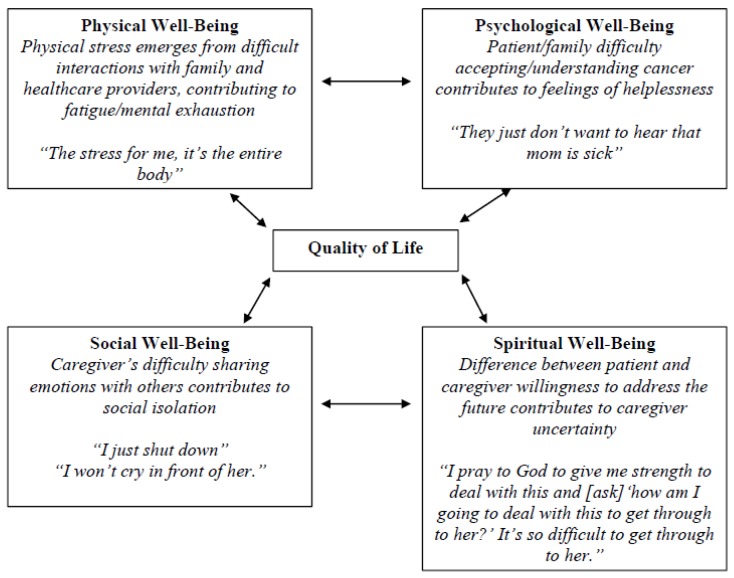
City of Hope Quality of Life model applied to caregiver communication burden.

Transcripts from communication coaching calls for 20 lung cancer caregivers were used for this analysis. The mean age of caregivers was 56.1 years. Most caregivers were Caucasian (80%), female (70%), and college-level educated (75%). The majority of caregivers were spouse/partner (65%). Table 1 shows a summary of caregiver characteristics. In the following section, we detail the thematic analysis of physical, psychological, social, and spiritual quality of life as it relates to caregiver communication burden. Figure 1 illustrates the QOL model with these themes.

### 3.1. Physical Well-Being

Physical well-being includes fatigue, sleep disruption, nausea, appetite (increase or decrease), and aches and pains from providing physical patient care. While the majority of caregivers described their own personal physical limitations due to illness or disability, they also described how communication burden was created by stress from difficult interactions. Stressful interactions contributed to feelings of fatigue and mental exhaustion. One caregiver described communication about her husband’s cancer with her daughter: “It’s mentally exhausting. She just stresses me out…When we get into it, it’s physically…I start shaking and I can feel my blood pressure go up. I have headaches.” Another caregiver shared that her blood pressure increases: “I lose my breath. When you take a deep breath and you kind of forget to take another breath…the stress for me, it’s the entire body.”

Talking with healthcare providers was acknowledged as a physically stressful event. Reflecting on prior experiences with a particular physician, one caregiver explained: “It was horrible for me because I get choked up and I couldn’t speak and I had heart palpitations.” Other caregivers shared that they were anxious about sitting down to talk about the diagnosis or prognosis (“I feel nervous, especially if the news is not as good as had hoped”) and doubted their “ability to communicate properly.”

### 3.2. Psychological Well-Being

Psychological well-being includes feelings of anxiety, depression, helplessness, difficulty coping, and fear. Feelings of helplessness surfaced for caregivers when the patient or a family member had difficulty accepting or understanding the severity of the cancer diagnosis and treatment side effects. In one instance, a caregiver described how her husband’s avoidance of cancer was problematic given her role as both a caregiver and mother: “We have a problem…he doesn’t want me to talk, to tell the kids. We haven’t told our children because he doesn’t want me to tell them…Do I just go ahead and do it and go behind his back and tell them, or do I let him have this wish?” A lack of opportunity to discuss cancer with others left caregivers feeling helpless. There was an acute awareness among caregivers that others were unable to engage in conversations about cancer. Caregivers recognized clear attempts by others to block, ignore, or avoid discussions about cancer. One caregiver explained his daughter’s unwillingness to talk about her mother’s illness:

“She just brushes it off and when you try to talk to her about it, she gets very frustrated about it because she doesn’t want to accept that her mother might possibly pass on due to cancer…So you really can’t talk…She just won’t hear it…She gets in this mood, so it’s something you don’t want to have to bring up.”

In response to his daughter’s reluctance to discuss the topic, this caregiver described reciprocally avoiding the topic in order to circumvent stress.

Communicating with children was identified as another form of communication burden as caregivers worried that talking about cancer would impact their child’s psychological well-being. One caregiver described wanting to achieve a delicate balance of addressing the topic and keeping the child informed, yet not causing alarm: “I have to be very accurate, but at the same time cushion it and not cause induced stress.” Another caregiver explained that she worried about the impact on her son: “Anything I say has immediate ramifications as to his mental well-being and this mood…and lasting ramifications.” The decision over whether or not to talk with children impacted psychological well-being for caregivers who often struggled with how to talk with kids about cancer:

“We don’t know what approach we should talk to them or even if we should not talk to them…If we were to talk to them, how are we going to approach how they are going to understand.”

Anxiety about communication caused caregivers to be mindful of how communication about cancer occurs with others, as one caregiver described: “It just took me awhile to think or how to approach what words I’m going to use to describe [her being sick]…my wife doesn’t even mention cancer…we always say illness.” Anxiety also triggered reflective thinking about the caregiver’s own communication performance and strategies for communication:

“It was just this continuous dialogue in my head after I’ve spoken with them…you know, I probably should have said or that I didn’t convey things properly…I have increasing anxiety afterwards…after the conversation that I didn’t convey my message properly.”

“They refuse to hear, see above my mom’s illness or they don’t bother to ask. I have to take the initiative and make the conversation and try to see how I could talk about it with them.”

### 3.3. Social Well-Being

Role adjustment, changes to relationships, leisure activities, and employment impact social-well being, sometimes leading to isolation. Difficulty sharing emotions and feelings about cancer added to communication burden and caregivers described withdrawing from others to avoid communication. One caregiver shared that he wanted to protect his wife by hiding his emotions: “I try to pretend I’m being tough (laughs) so I don’t cry in front of her…I just swallow it…my emotions.” Another caregiver summarized his family’s indirect nature about his wife’s cancer: “We really don’t discuss that [cancer] much unless she has an episode or something while we are all together, but basically we don’t bring it up as to how serious it is.”

Emotional distancing was also illustrated as a way of avoiding communication and sharing feelings. Separating from others to avoid communication about cancer was shared by one caregiver: “I just shut down and change the subject and find a reason to leave the conversation or leave completely.” A caregiver recalled learning about the initial cancer diagnosis from a radiologist and being asked to relay the news to her mother. In thinking about sharing this news with her mother, she described: “I wanted to disappear and go into a black hole and not think about it…because my mom was still in the wheel chair and my dad with her…I didn’t even want to see my mom. I was completely shut down.” Another woman explained: “Actually right now we are avoiding. We don’t talk to most family…a lot of my further family, I can’t talk about him [patient]. I can’t discuss it.” Engaging in social isolation was a way of navigating communication burden.

### 3.4. Spiritual Well-Being

Feelings of uncertainty, a search for hope and meaning, and religiosity all comprise spiritual well-being. Communication burden was created when there was a difference between patient and caregiver willingness to address feelings of uncertainty and explore the meaning of illness. One caregiver shared that “talking about the cancer outcome as far as the quality of life or what’s to come…that’s what makes it more challenging…talking about the cancer and what’s the future.” Caregivers felt nervous communicating with the patient about the disease and “not wanting to go in certain directions with the conversations.” Fear of upsetting the patient was a concern for one caregiver: “I know he will get disappointed. He will get upset. I don’t want him to go through that. Emotionally, for me, it would be hard for…to let him know about those [sic] stuff.” When asked to describe the biggest barrier to communicating better, another caregiver replied, “Not knowing, the unknown, not knowing what’s going to happen…because right now it’s arrested somewhat but it has not disappeared.” “It’s hard for me to talk to him (patient).” Another caregiver articulated the same for his family: “I have a level of acceptance that is relatively high, and they have a level of acceptance that is relatively low, and it makes it difficult to talk.”

On the other hand, there was an absence of communication burden described by caregivers with spiritual beliefs. Caregivers described that a sense of spirituality gave them strength to have conversations about cancer with others: “I have to work myself up to it (talking with family) and talk with my higher power about how I am going to approach this next conversation.” Praying together was also identified as a way of engaging in conversations about cancer and the future. Notably, a patient’s deep religious beliefs were characterized as enabling open conversation about illness: “I think the strongest support has been our whole family’s strong spiritual belief.”

## 4. Discussion

The QOL model is a theoretical framework that offers a way to understand the impact of cancer caregiving. The model outlines four domains as the components for caregiver assessment. Our aim in this study was to describe how communication burden impacts caregiver quality of life in each domain. An extensive meta-analyses has shown that interventions with family caregivers of cancer caregivers result in improved quality of life [12], and the analyses presented here extends the use of the QOL model to understand how communication negatively impacts caregiver quality of life, thus informing the need for future interventions to include caregiver assessment and communication skills training content.

Findings from this study confirm that caregiver quality of life is impacted by stress about communication across all domains. Future intervention content should address the caregiver’s unique communication role by (a) including a baseline assessment of caregiver communication skills; (b) providing intervention materials and content that teach new communication skills, especially for sharing difficult news with others; and (c) measures of communication that assess the efficacy of teaching communication to family caregivers. For example, a caregiver communication skills training program may be organized by the quality of life domains and include ways to take practice self-care (physical well-being), suggestions for how to share concerns or feelings (psychological well-being), ways to ask for help from other family members and healthcare providers (social well-being), and example questions to ask to explore life meaning and purpose (spiritual well-being).

Initiating conversations was identified in this study as a critical role for caregivers that caused stress and burden, impacting their quality of life. Among cancer caregivers, relationship quality between patient and caregiver is an important contextual factor that aids caregiver coping and impacts the caregivers’ stress process [13]. In the present study, caregivers reported a clear understanding of the need to bring up topics in order to carry out caregiving duties and how this would evoke stress. When conversations about caregiving and patient care were blocked by the patient or family members, this created communication-related burden that impacted caregiver quality of life. Future intervention research should include attention to the caregiver’s role in sharing information with other family members and friends about the patient’s cancer. Caregiver interventions that include communication skills building have the potential to decrease caregiving burden related to role changes and improve caregiver quality of life [14].

Prior research has documented that the lung cancer experience has a profound impact on the well-being of both patient and family caregiver and is largely influenced by communication within the family environment [15,16]. Lung cancer caregivers attempt to protect the patient and maintain hope by avoiding discussions about the diagnosis and illness trajectory [17]. The desire to protect each other results in topic avoidance between caregiver and patient, impacting communication with other family members and healthcare providers. Communication avoidance from patient and family members was reported by caregivers in this study, impacting caregiver psychological and spiritual quality of life. Communicating with children about cancer was also identified as a challenging caregiving task characterizing communication burden, and future research should address the need for caregivers to learn new skills for relaying and sharing news to children within the family structure.

Research on adult children caring for a parent with lung cancer found that emotional overload was common and that negative emotions resulted in communication avoidance [5]. Patient anxiety is associated with family’s avoidance of cancer communication [18,19] and can be triggered by conflict with family members [20]. Disagreements between patient and caregiver are also associated with depression [3] and can impact the caregiver’s ability to provide care [15]. Findings from this study demonstrate that caregivers need skill training in how to navigate topic avoidance, geographic distance of key family members, and changing family relationships [5].

### Limitations

During the communication coaching call, not all caregivers commented on all QOL domains. Thus, this analysis may not have identified the proportion of caregivers who experienced a particular QOL domain communication issue. Among caregivers in this study, psychological well-being was impacted most by communication burden. However, this study is limited by a small sample size and a predominant sample of college-educated, Caucasian caregivers.

## 5. Conclusions

The findings of this study indicate that communication-related burden, as perceived by lung cancer caregivers, impacts all four QOL domains. When communication avoidance is apparent, it is important to address caregiver communication burden. Content for cancer caregiving interventions should include communication skill building, including strategies for self-care.

## Figures and Tables

**Table 1:** Caregiver characteristics.

Characteristic	*n* = 20 (%)
Age, mean (range)	56.1 (35–76)
**Gender**	
Female	14 (70%)
Male	6 (30%)
**Relationship to Patient**	
Spouse/Partner	13 (65%)
Adult child	6 (30%)
Other	1 (5%)
**Education**	
Secondary School	5 (25%)
College	15 (75%)
**Hispanic Ethnicity**	
Yes	4 (20%)
No	16 (80%)
**Race**	
African-American	1 (5%)
Asian	3 (15%)
Caucasian	16 (80%)
**Employment Status**	
At least 32 h	11 (55%)
Retired	7 (35%)
Self-employed	2 (10%)
**Household Income**	
$10,000 or less	1 (5%)
$20,001 to $30,000	2 (10%)
Greater than $50,000	14 (70%)
Prefer not to answer	3 (15%)
